# Variation in plant leaf traits affects transmission and detectability of herbivore vibrational cues

**DOI:** 10.1002/ece3.6857

**Published:** 2020-09-30

**Authors:** Estefania Velilla, Jernej Polajnar, Meta Virant‐Doberlet, Daniel Commandeur, Ralph Simon, Johannes H. C. Cornelissen, Jacintha Ellers, Wouter Halfwerk

**Affiliations:** ^1^ Department of Ecological Science Vrije Universiteit Amsterdam Amsterdam The Netherlands; ^2^ National Institute of Biology Ljubljana Slovenia

**Keywords:** biotremology, leaf traits, plant‐borne vibrations, plant‐herbivore interactions, transmission

## Abstract

Many insects use plant‐borne vibrations to obtain important information about their environment, such as where to find a mate or a prey, or when to avoid a predator. Plant species can differ in the way they vibrate, possibly affecting the reliability of information, and ultimately the decisions that are made by animals based on this information. We examined whether the production, transmission, and possible perception of plant‐borne vibrational cues is affected by variation in leaf traits. We recorded vibrations of 69 *Spodoptera exigua* caterpillars foraging on four plant species that differed widely in their leaf traits (cabbage, beetroot, sunflower, and corn). We carried out a transmission and an airborne noise absorption experiment to assess whether leaf traits influence amplitude and frequency characteristics, and background noise levels of vibrational chewing cues. Our results reveal that species‐specific leaf traits can influence transmission and potentially perception of herbivore‐induced chewing vibrations. Experimentally‐induced vibrations attenuated stronger on plants with thicker leaves. Amplitude and frequency characteristics of chewing vibrations measured near a chewing caterpillar were, however, not affected by leaf traits. Furthermore, we found a significant effect of leaf area, water content and leaf thickness—important plant traits against herbivory, on the vibrations induced by airborne noise. On larger leaves higher amplitude vibrations were induced, whereas on thicker leaves containing more water airborne noise induced higher peak frequencies. Our findings indicate that variation in leaf traits can be important for the transmission and possibly detection of vibrational cues.

## Graphical abstract

Specific leaf traits such as leaf thickness, leaf area, and water content can influence the transmission and possibly the detectability of herbivore‐induced vibrational chewing cues. Our results are particularly interesting in light of the arms race between plants, their herbivores, and the natural enemies of plant herbivores where vibrations have been demonstrated to mediate orientation towards prey (vibrotaxis) by insect parasitoids.

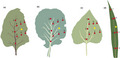

## 1 INTRODUCTION

Animals can extract and use a vast amount of information from their biotic and abiotic environment. This information can be encoded by different sensory stimuli like sounds, odors, or vibrations and may guide animals in many important life‐history decisions (Dall et al., 2005; Stevens, 2013). Biotic environmental information such as incidental cues (e.g., prey cues) or purposeful signals (e.g., mating signals) are used to decide whom to mate with, where to search for food, or when to adopt predator avoidance behavior (Dall et al., 2005; Stevens, 2013). Additionally, abiotic environmental information can warn animals of changes in their habitat, such as the sound cues of upcoming rain or visual lightning cues of an approaching storm (Geipel et al., 2019; O’Connell‐Rodwell et al., 2001). However, sensory conditions often vary across time and space, which can have important consequences for the reliability and usefulness of environmental information (Dall et al., 2005; Stevens, 2013).

Reliable transmission and perception of signals and cues can be affected by habitat‐dependent factors such as environmental complexity and noise levels (Boncoraglio & Saino, 2007; Brumm & Slabbekoorn, 2005; Elias & Mason, 2014; Mortimer, 2017; Morton, 1977; Richards & Wiley, 1980; Tobias et al., 2010). Environmental or anthropogenic background noise has also been documented to affect the perception of signals and cues in a wide range of animals and for a wide range of contexts (Brumm & Slabbekoorn, 2005; Ord et al., 2007; Schaub et al., 2008; Wu & Elias, 2014).

A clear characterization of the sensory environment is therefore crucial for understanding its effects on the transmission and perception of environmental signals and cues. However, characterizing the sensory environment can be challenging because it often entails a three‐dimensional, complex, and large space through which information must travel.

Substrate‐borne vibrations traveling through soil, rocks, and plants are used as a sensory stimulus by hundreds of thousands of invertebrates and many vertebrates as well (Cocroft et al., 2014; Cocroft & Rodriguez, 2005). In particular on plants, vibrations travel relatively short distances in a one or two‐dimensional plane and their active range is determined primarily by the shape and size of particular plant species (Čokl et al., 2005; Mazzoni et al., 2014; Michelsen et al., 1982). Plants are highly complex structures and exhibit large variability in traits that can affect the transmission of vibrational signals and cues (Mankin et al., 2018). Within‐species comparisons have shown that vibratory transmission properties vary with plant age, turgidity, and development and disappearance of organs and tissues (Bell, 1980). Also differences among plant species may affect the production, transmission and perception of vibratory signals and cues, leading to important consequences for the animals that live on them (Cocroft et al., 2006; Joyce et al., 2014). Signal variability can affect the detection and proper assessment of the signal or cue by the receiver, possibly decreasing mate attraction or prey localization, as has been shown in spiders (Gordon & Uetz, 2011; Rosenthal et al., 2019). For that reason, knowledge on the interspecific variation in transmission and sound‐absorbing properties of plant tissues is important (Gagliano et al., 2012, 2017; Schoner et al., 2016). For example, leaf shape and leaf thickness may affect transmission of vibrations, or influence absorption of airborne sounds, and consequently interfere with detection of vibratory signals and cues.

In this study, we aimed to understand how leaf traits affect the production, transmission and detectability of chewing vibrations. We focused on the vibrations produced by the beet armyworm *Spodoptera exigua* (Hübner; Lepidoptera, Noctuidae) caterpillar chewing on the leaves of different plant species. Chewing vibrations induced by foraging insects are known to travel throughout the herbaceous plant tissues and can be picked up by a wide range of organisms, including predators and parasitoids (Meyhofer & Casas, 1999), and even the plant itself (Appel & Cocroft, 2014). We chose *S. exigua*, because of its diverse diet, which allowed us to compare vibrational chewing cues on four different host plant species with divergent leaf traits including eudicot species: sunflower *Helianthus annuus*, cabbage *Brassica oleracea var. Capitata*, beetroot *Beta vulgaris*, and the monocot corn *Zea mays*.

We designed a set of experiments to quantify the extent of intra‐ and interspecies variability in the production and transmission and detectability of chewing‐induced vibrations along leaves. We predicted that plant species would have an influence on cue attenuation and absorption of environmental noise, and that the differences would be related to physical leaf traits.

## 2 METHODS

### 2.1 Animal rearing

The beet armyworm *S. exigua* is a polyphagous insect pest with a worldwide distribution and is considered a serious pest of vegetables, field, and flower crops. *Spodoptera exigua* was reared at 26°C ± 1°C and 80% relative humidity on a 12:12 hr (L:D) photoperiod. Larvae were fed with a corn‐based artificial diet, and adults were given 10% sucrose solution. Mating was facilitated by placing a male and a female moth in a plastic round container with a mesh cloth sealing the top. The mesh cloth was used as a surface on which eggs were laid. Eggs were collected by removing the cloth and cutting and placing the sections of the cloth containing eggs on diet‐filled petri dishes. As eggs hatched, larvae developed on these diet‐filled petri dishes. The life cycle of *S. exigua* under our rearing conditions was completed between 25–30 days and included 5 instars. Each instar transition took between 3 and 5 days.

### 2.2 Plant rearing and measuring of plant leaf traits

Four plant species were used for this experiment: sunflower *H. annuus*, cabbage *B.oleracea var. Capitata*, beetroot *B. vulgaris*, and corn *Z. mays*. Seeds were bought from commercial companies: Seedo and 123Zaden (The Netherlands). Plants were reared in growing chambers at 20°C–25°C with an 8:16 hr (L:D) photoperiod and with 70% relative humidity. Quartz sand was used instead of potting soil to control for any variation in soil quality that could influence plant traits. A fixed amount of 50% Hoagland's solution was provided every second day. Amount changed gradually with the growth of the plants (~10–100 ml). Plants that were between 6 and 8 weeks postgermination were used in the experiments.

**Figure 1:**
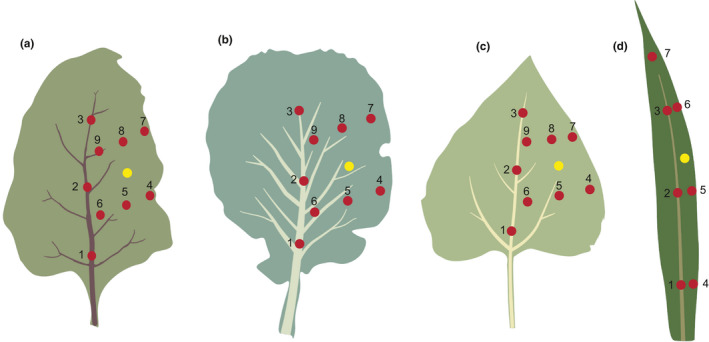
Schematic illustration of selection of points on a leaf for leaf trait measurements for the different plant species. (a) Beetroot, (b) cabbage, (c) sunflower, (d) corn. Points 1, 2, and 3 covered the main vein, whereas other points were positioned on soft tissue. We did not consider whether a point on soft tissue covered a smaller vein or capillary. The yellow point represents the source of stimulation for the transmission experiment. Due to the very different shape of corn leaves (d), the point amount and distribution was different

We measured four leaf traits in the laboratory: leaf area (cm^2^), leaf mass (g), leaf thickness (mm), and punch force (N) following the handbook for standardized measurement of plant functional traits (Pérez‐Harguindeguy et al., 2013). Leaf area was measured by scanning the leaves, and subsequently measuring the area with the program ImageJ. The dry weight was calculated by first drying the harvested leaves for at least 48 hr in a 70°C oven, and then weighing them. Dry weight and leaf area were used to calculate the specific leaf area SLA (cm^2^/g) (one‐sided area of a fresh leaf, divided by its dry weight). Leaf thickness was measured with a manual caliper on nine locations on the leaf, seven for corn (Figure 1). Punch force was measured as the maximum (i.e., pulse) force needed for a 1 mm diameter blunt needle to puncture the leaf, which was clamped tightly on either side of the puncturing spot using a Mecmesin Ultratest Newton meter with Force Gauge AFG 1000‐N (Mecmesin, Broadbridge UK). These measurements were done on the same nine locations within eudicot leaves and in, the same seven locations for corn. The measuring points one, two, and three covered the main vein, and the points four, five, six, seven, eight, nine the soft tissue (Figure 1). The fresh weight and dry weight were used to calculate the fresh weight to dry weight ratio, which we interpreted as the water content.

We measured leaf traits of two sets of plants. The first set (*n* = 101) consisted of plants paired up with the plants used in the recordings of caterpillar chewing vibrations (chewing experiment). This paired design allowed us to measure leaf traits in plants undamaged by chewing while being representative of the leaf traits of chewed plants, but relied on the assumption that paired plants used to measure traits represent the leaf trait variation of the plants on which chewing measurements were done. Although this assumption may not have been met for all pairs, it still seems reasonable compared to the alternative design which would involve measuring leaf traits and vibrations on the same leaf, given that chewing behavior could be affected by prior measuring of the traits (e.g., damage by punch force tests could have elicited secondary metabolites), and trait measurement could have been affected by foraging (e.g., loss of mass or strength after herbivory attack). The second set of plants (*n* = 40) corresponds to the transmission experiment, described in a section below. In this case, there was no confounding effect of herbivory, and we therefore used the same plants to measure vibrations and leaf traits.

### 2.3 Recordings of caterpillar chewing vibrations

The purpose of these recordings was to investigate the effect of variation in leaf traits on the production of caterpillar chewing vibrational cues. We first made recordings of caterpillars chewing on different plant species, and we then related the amplitude and frequency characteristics of those recordings to variation in leaf traits. A custom‐built wooden box (90 cm × 60 cm × 60 cm) with a Plexiglas top was used to reduce airborne noise during recording of the chewing vibrations. The box was lined with noise absorbing foam and was placed on a vibration reduction marble table with passive suspenders to minimize substrate‐borne noise of the building. The airborne noise amplitude inside the box at the location of the plant was approximately 35 dB (A) measured with an Extech SDL600 sound level meter (set to fast and max).

Caterpillar chewing was recorded using a Laser‐Doppler Vibrometer (LDV; Polytec PDV‐100, set to 5 or 20 mm s^−1^ V^−1^, sampling rate 22 kHz). The output of the laser was acquired using a TASCAM DR‐60D MKII audio recorder (44,1 kHz, 16‐bit resolution). The recording level of the Tascam was set so that there would not be overload of the signal. A reference signal using the same recording level was made against the vibration‐isolation table after each recording to allow calculation of absolute amplitude. A reference signal can be made by generating a sinusoidal test signal of 2.80 V (RMS) with a frequency of approximately 1 kHz by setting the laser service mode to Output = Full (Polytec PDV‐100 user manual, section 5). We recorded this test signal on the table on which we placed our plants with caterpillars and we recorded it with the TASCAM with the exact same recording settings used per recording of caterpillar chewing vibrations. A single plant was placed inside the noise‐isolation box, and a free‐moving caterpillar was placed on a leaf of the plant. The caterpillar was always dropped on the middle of the leaf, but it foraged freely and therefore the foraging position changed per trial. A piece of reflective tape placed central on the measuring side of the leaf was used to enhance reflection of the laser beam. Recordings started always around the same time of day (~13:00). A recording started when a caterpillar started eating, and the recording lasted 30 s. The distance from feeding site to the measurement site (reflective tape) was noted. The distances ranged from 0.50 to 33.3 mm, with a mean ± *SD* distance of 12 ± 8.7 mm. Individual plants and caterpillars were used only once for each recording. Recordings were done throughout three larval stages of the animals (L2‐L4/5). Before the recordings, caterpillars were weighed and allowed to acclimatize to the experimental chamber. We had 19 replicates for cabbage, 24 for beetroot, 18 for sunflower, and 16 for corn. However, eight recordings were too low in amplitude and the chewing vibrations were not distinguishable from the background noise levels. Therefore, our total sample size was reduced to 69 caterpillar–plant combinations.

### 2.4 Transmission experiment

To test which leaf traits affected the transmission of caterpillar chewing cues among plant species, we conducted a vibrational playback experiment. The purpose of this experiment was to measure the transmission of a synthetic vibratory signal throughout the leaves of the different plant species we used during the chewing vibrations recordings. We then related these transmission measurements to the different leaf traits. Using a Brüel & Kjær mini‐shaker Type 4,810, we played back a 2‐min frequency sweep starting at 20 Hz and ending at 2 kHz. The playback was corrected for the frequency response of the shaker to ensure constant velocity at all frequencies and adjusted to the RMS amplitude level of chewing vibrations recorded in caterpillar trials. This adjustment was necessary because the frequency response of the mini‐shaker is highly nonlinear in the low frequencies (<100 Hz), biasing the playback to the frequencies >100 Hz in a noncorrected format. Therefore, by correcting our playback file to the frequency response of the shaker, we made sure all frequencies were equally represented. We used two LDVs, one to register vibrations next to the source (Figure 1) and the other on the nine different points on the leaf, seven for corn (Figure 1). These points were the same points that were used for measuring leaf thickness and punch force (Figure 1). Furthermore, we recorded the sweep on the adjacent leaf to test relative energy loss during transmission.

Vibrations were transferred via a rod mounted on the shaker and attached with Blue Tack adhesive to the underside of the leaf on a point in the center of the soft tissue, not touching the main vein, between points five and eight (Figure 1). The distance between each point and the stimulus was noted. The distances ranged from 1 to 20 mm. We tested five plants per species.

### 2.5 Airborne noise exposure experiment

To test whether amplitude of vibrations induced by airborne noise was affected by leaf traits, we did an acoustic noise playback experiment with white noise (0.1–20 kHz) (Rebar et al., 2012). Using a Behringer MPA40BT speaker positioned 60 cm from the plant, we played back 10 s of white noise at 70 dB (A) measured at the position of the leaf with an Extech SDL600 sound level meter (set to fast and max) and recorded with the LDV on three points (points 1:3, Figure 1). Using an LDV, we recorded the vibrations induced by the airborne noise playback. We tested five plants per species.

### 2.6 Analysis of vibratory measurements

Chewing recordings were first filtered with a 100 Hz high pass filter in R version 3.3.1 (R Core Team, 2016), run in the RStudio interface (RStudio Team, 2015) with the function “fir” from the Seewave package version 2.1.4 (Sueur et al., 2008). Recordings were filtered to remove high amplitude, low‐frequency background building noise. Using Raven Pro 1.5 software (Cornell Lab of Ornithology 2017), we selected ten chewing events per recording (see Figure S1 in Appendix S1 for an example). We measured root mean square (RMS) amplitude from the waveform, and first and third quartiles. Peak frequency was taken from the spectrum (sampling frequency: 44,100, window type: “Hanning,” window size: 1,024, overlap: 50). All measurements were done on the filtered recordings. Reference recordings were filtered in the same way as chewing recordings. RMS measurements from the reference recordings were used to calculate absolute RMS amplitude (mm/s) of chewing events. To calculate absolute RMS amplitude, we used the following formula:

$$\text{RMS amp}\left(\frac{\text{mm}}{s}\right)=\left(\frac{\text{RMS}\left(\text{measurement}\right)}{\text{RMS}\left(\text{reference}\right)}*2.80\right)*\text{LDV vel scaling setting}$$

The value of 2.80 represents the RMS amplitude (in Volts) output of the LDV, and the LDV velocity scaling setting was either 5 or 20 mm s^−1^ V^−1^ (Polytec PDV‐100 user manual, section 5).

### 2.7 Analysis of vibratory sweep and acoustic noise playbacks

**Figure 2:**
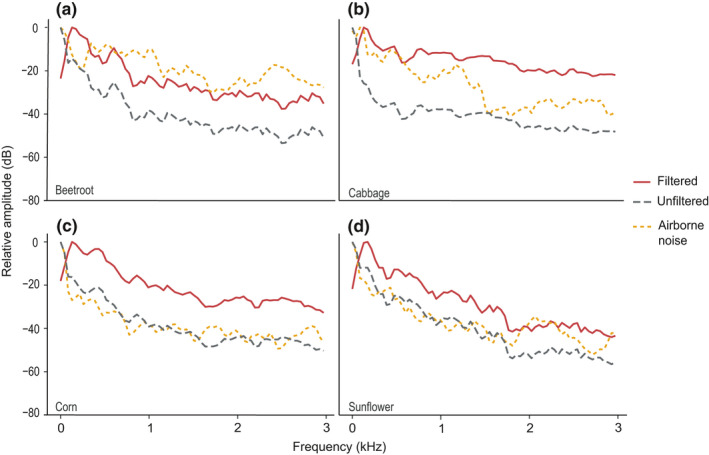
Normalized averaged amplitude spectra of the unfiltered and filtered chewing recordings, and of the airborne noise stimuli on (a) beetroot, (b) cabbage, (c) corn and (d) sunflower. Sampling rate: 44,100 Hz, window type: Hanning, window size: 1,024, overlap: 50, The dark gray dotted line represents the unfiltered recordings, and the yellow dotted line the airborne noise. The spectra show that most of the chewing cue remains unchanged after filtering

The sweep recordings were high‐pass filtered in the same way as the chewing recordings. The main frequency range of chewing recordings was determined by plotting frequency spectrum of a representative recording per plant species (Figure 2). We determined RMS amplitude of reference recordings in Raven Pro, which were made for every recorded point, including the ones on the adjacent leaves. Attenuation (dB) was calculated for every measurement point of every individual plant using the following formula:

$$20\text{Log}10=\frac{\text{RMS}\left(\text{measurement}\right)}{\text{RMS}\left(\text{reference}\right)}$$

We also calculated peak frequency from the spectrum (sampling frequency: 44,100, window type: “Hanning,” window size: 1,024, overlap: 50). Because we did not measure transmission on the stem, we decided to use the midvein points and the adjacent leaf as a proxy for transmission. Hence, our statistical analyses not only explore differences in the average amplitude change (dB) and mean peak frequency (Hz) measurements across all points on the same leaf, but also of the midvein points, as well as transmission via the stem to the nearest leaf.

Noise recordings were also high‐pass filtered and RMS amplitude (dB) and peak frequency (Hz) measurements obtained with Raven Pro. All recordings were normalized in R by dividing them by the maximum amplitude of the loudest recording.

### 2.8 Statistical analyses

Statistical analyses were done with R version 3.3.1 (R Core Team, 2016), run in the RStudio interface (RStudio Team, 2015).

### 2.9 Differences in leaf traits

To evaluate differences in leaf traits across plant species, we fitted linear mixed effects models from the package “Lme4” (Bates et al., 2015). Because leaf traits were measured on two batches of plants (the batch for the chewing experiments and the batch for the transmission experiment), we modeled “experiment” as a random effect with a random intercept. For punch force and leaf thickness, we also included point on the leaf as a random effect because we measured these traits on nine different locations (seven on corn) on the leaf. We fitted a model per leaf trait with plant species as predictor. By means of a Tukey test from the statistical package “lsmeans” (Lenth, 2016), we conducted pairwise comparisons among species.

### 2.10 Differences in RMS amplitude and mean peak frequency measurements from chewing cues across plant species

We fitted two multiple regression models with Gaussian distribution to evaluate differences in RMS amplitude (mm/s) and mean peak frequency (Hz) measurements across plant species. Caterpillar weight (g) and distance from chewing to measuring point (mm) were included as covariates. Amplitude was log transformed. We calculated pairwise comparisons among species by means of a Tukey HSD test.

### 2.11 Effect of leaf traits on RMS amplitude and mean peak frequency measurements from chewing vibrations

To determine which leaf traits contributed to explaining variance in RMS amplitude and peak frequency measurements from chewing vibrations, we followed an information‐theoretic approach (Burnham et al., 2011). We used the R package “MuMIn” (Barton, 2009) to evaluate 32 linear mixed effects candidate amplitude and frequency models. The linear mixed effects models were produced with the package “Lme4” (Bates et al., 2015). The leaf traits included in the RMS amplitude and peak frequency candidate models were: SLA (cm^2^/g), leaf thickness (mm), and water content (g/g). We left out the traits, punch force, leaf area, and leaf mass because of high collinearity between punch force and leaf thickness, and between leaf mass and SLA, and leaf area and SLA. Additionally, the models contained plant species as a random effect with a random intercept. Using Akaike's information criterion for smaller sample sizes (AICc), we assessed the explanatory value of the candidate models comparing them by their ΔAICc = (AIC_i_ – min AIC). We considered all models with a ΔAICc_i_ ≤ 4. Akaike weights (*w*_1_) were computed to determine the probability that a model was the best model. We then calculated the model average estimates with the subset of models that had a ΔAICc_i_ ≤ 4. Additionally, we calculated 95% confidence intervals.

For this analysis, we used the mean leaf thickness across six of the nine measured points. These six points corresponded to the soft tissue of the leaf (points 4–9, Figure 1) and excluded the points along the midvein (1–3, Figure 1). We decided to exclude the midvein points because the caterpillars we tested only foraged on soft plant tissue and never chewed from the midvein. Amplitude was log‐transformed in the amplitude model.

### 2.12 Effect of leaf traits on amplitude attenuation and mean peak frequency measurements from the vibrational sweep playbacks

To test the effect of leaf traits on RMS amplitude and mean peak frequency measurements of the vibrational sweep playback, we followed the same approach as above. We evaluated 8 linear mixed effects candidate amplitude models and 8 frequency models. The candidate models contained exactly the same leaf traits as in the analysis above. We repeated this process to test the effect of leaf traits on amplitude and frequency measurements taken from the adjacent leaf.

### 2.13 Effect of leaf traits on amplitude and mean peak frequency induced by airborne noise playbacks

Once again, we followed an information‐theoretic approach (Burnham et al., 2011). We evaluated 16 amplitude and frequency candidate models. In this analysis, we replaced SLA by leaf area because leaf area is more likely to affect RMS amplitude measurements. We calculated the averaged estimates for the models that were within ΔAICc_i_ ≤ 4 of each other.

All figures were created using the library “ggplot2” version 3.1.0 (Wickham, 2016).

## 3 RESULTS

### 3.1 Differences in leaf traits

**Figure 3:**
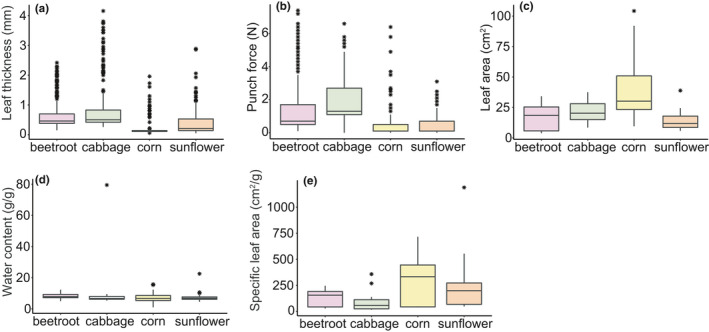
Boxplots showing variation in leaf traits across plant species. This graph combines the plants of both batches of experiments (chewing experiment, *n* = 101 and transmission experiment, *n* = 40). (a) leaf thickness is highest for cabbage and lowest for corn, (b) punch force is also highest in cabbage and beetroot and lowest in corn and sunflower, (c) leaf area was highest in corn, (d) water content was also highest in corn, (e) specific leaf area was highest in corn and sunflower, and cabbage had the lowest levels of specific leaf area. The effect of plant species was tested with a linear mixed effects model that included experimental batch as a random effect. Plant species had a significant effect on all traits. The interquartile range was taken as the range from 0 to 25th percentile. From the mean, the whiskers show the highest and the lowest value within 1.5 times the interquartile range

All leaf traits differed significantly across species, except for water content (Lmm, SLA (log transformed), *F* = 65.54, *p* < .01; punch force (square root transformed), *F* = 360.71, *p* < .01; leaf thickness (log transformed), *F* = 487.84, *p* < .01; leaf area (log transformed), *F* = 39.103, *p* < .01; water content, (log transformed), *F* = 2.0498, *p* = .1097, Figure 3). In both experiments, sunflower and corn had the thinnest leaves and the lowest punch force, whereas cabbage and beetroot had the highest leaf thickness and punch force. Although leaf traits displayed the same trends among plant species in both experiments, they differed largely between experiments, possibly due to a mismatch in development at the time of the experiments in spite of very similar growing environments and treatment.

### 3.2 Amplitude and mean peak frequency measurements from chewing vibrations differ across plant species

**Figure 4:**
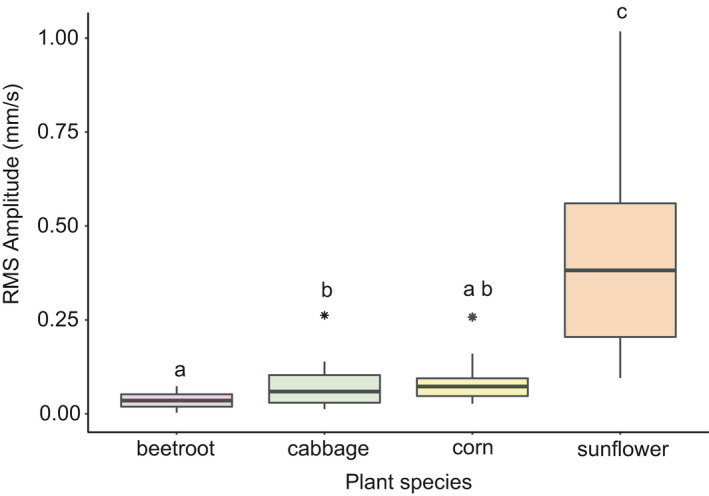
Boxplots showing the variation in RMS amplitude (mm/s) of chewing vibrations on the different plant species. The letters indicate significant pairwise differences (*p* < .05) calculated by a Tukey HSD post hoc test. The interquartile range was taken as the range from 0 to 25th percentile. From the mean, the whiskers show the highest and the lowest value within 1.5 times the interquartile range

Chewing vibrations recorded from different plant species differed in amplitude (Lm, *F* = 40.795, *p < *.01). We recorded the highest amplitude vibrations on sunflower and the lowest on beetroot (Figure 4). All plant species differed significantly in amplitude measurements from each other, except cabbage and beetroot, and cabbage and corn (Figure 4). Mean peak frequency differed significantly only in the cabbage–beetroot comparison (*p = *.02, Figure 4).

### 3.3 Effect of leaf traits on RMS amplitude and mean peak frequency measurements from chewing vibrations

**Figure 5:**
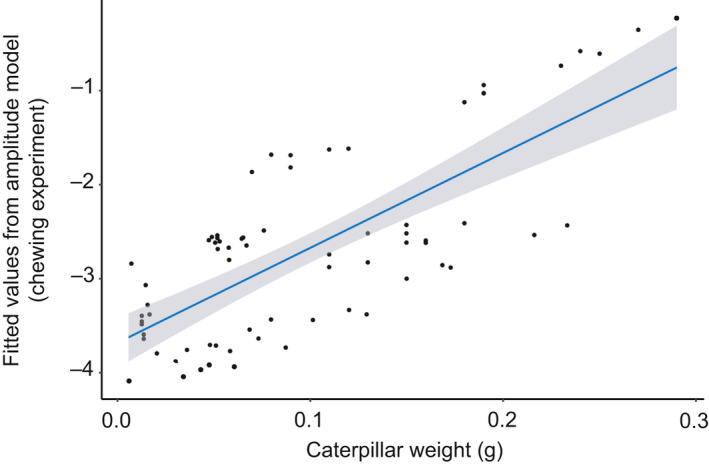
Fitted values from the amplitude model (chewing experiment) explaining variation in amplitude of caterpillar chewing cues plotted against caterpillar weight. The amplitude model contained the leaf traits: leaf thickness, specific leaf area and water content. Additionally, plant species and experimental batch were included as random effects with a random intercept. Amplitude increases with the increase in caterpillar weight. The blue line represents the linear relationship between the fit of the model and leaf thickness

None of the leaf traits we included in our candidate models significantly affected amplitude measurements. Although leaf thickness appeared in the top models (Appendix S1, Table S1), it did not have a significant effect on amplitude (Table 1). Moreover, caterpillar weight had a highly significant positive effect on amplitude (Figure 5, Table 1).

**Table 1:** Conditional averaged estimates from candidate models testing the effect of leaf traits on the amplitude and peak frequency measurements from caterpillar chewing vibrations

Parameter	Estimate	*SE*	*z* value	*p* value	95% CI	
					Lower	Upper
Log (amplitude) ~						
Caterpillar weight (g)	6.87005	1.3485	5.001	<.001	4.178073	9.5635359
Leaf thickness (mm)	−1.10835	0.6926	1.535	.125	−2.466768	0.2996736
Peak frequency ~						
Caterpillar weight (g)	−185.389	137.411	1.349	.177285	−454.708909	83.9307663
Distance (cm)	−1.986	1.073	1.851	.064227	−4.090220	0.1173903
Leaf thickness (mm)	61.322	61.143	1.003	.315891	−58.515019	181.1594401
Water content (g/g)	3.238	3.934	0.823	.410485	−4.472846	10.9488033

Note

All models within 4 AICc were considered. None of the leaf traits we measured significantly affect RMS amplitude or peak frequency measurements from chewing vibrations

Leaf thickness and water content appeared in all the models predicting variation in peak frequency of chewing vibrations (Appendix S1, Table S1). However, neither of these had a significant effect on peak frequency measurements (Table 1). Caterpillar weight and distance from chewing to recording point also appeared in the top models, but they also did not have a significant effect on peak frequency measurements (Table 1).

### 3.4 Effect of leaf traits on amplitude attenuation and mean peak frequency measurements from the vibrational sweep playbacks

**Figure 6:**
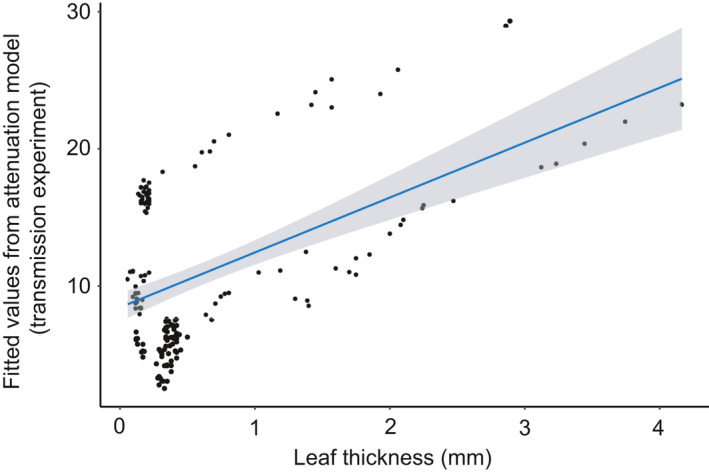
Scatterplot showing the fitted values from the attenuation of the vibrational sweep playback (transmission experiment) plotted against leaf thickness, which was the trait that significantly affected attenuation. The attenuation model contained the leaf traits: leaf thickness, specific leaf area, and water content. Additionally, point on the leaf nested in plant species was included as a random effect with a random intercept. Attenuation is higher in thicker leaves. The blue line represents the linear relationship between the fit of the model and leaf thickness

All the leaf traits we included in our candidate models appeared in the top models explaining variation in amplitude attenuation from the vibratory sweep playback (Appendix S1, Table S2). However, only leaf thickness had a significant effect (*β* = 4.7843, *p* < .01), with thicker leaves leading to higher attenuation (Figure 6, Table 2). For the frequency model, all leaf traits appeared in the top models explaining peak frequency measurements (Appendix S1, Table S2), though none of the traits had a significant effect, and the 95% confidence intervals largely overlapped zero (Table 2).

**Table 2:** Conditional averaged estimates from candidate models testing the effect of leaf traits on the attenuation and peak frequency measurements from vibrational sweep playbacks

Parameter	Estimate	*SE*	*z* value	*p* value	95% CI	
					Lower	Upper
Attenuation ~						
Leaf thickness (mm)	4.7843	0.7994	5.985	<.001	3.2175844	6.3510541
SLA (cm^2^/g)	−0.1015	0.1728	0.587	.5570	−0.4401452	0.2371717
Water content (g/g)	0.2314	0.7696	0.301	.7636	−1.2770046	1.7398850
Peak frequency ~						
Leaf thickness (mm)	0.4012	6.1126	0.066	.948	−11.579329	12.3817850
SLA (cm^2^/g)	0.1992	0.3808	0.523	.601	−0.547168	0.9455917
Water content (g/g)	1.2049	3.7212	0.324	.746	−6.088454	8.4983418

Note

All models within 4 AICc were considered.

When we tested the effects of the same leaf traits on amplitude attenuation of the vibratory sweep recorded on the adjacent leaves, the best model turned out to be the null model, which contained only the random effects (point nested in species) and none of the leaf traits. The same was the case for the frequency model of the adjacent leaves (Appendix S1, Tables S3 and S4). None of the traits explained significant variation in amplitude attenuation on the adjacent leaf.

### 3.5 Effect of leaf traits on amplitude and mean peak frequency induced by airborne noise playbacks

**Figure 7:**
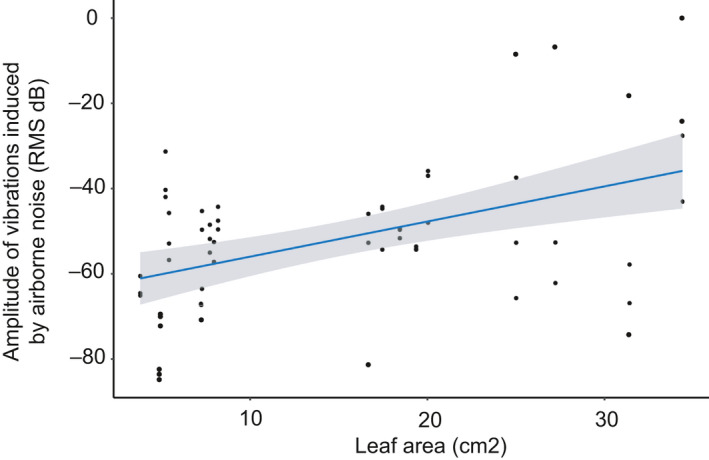
Fitted values from the amplitude model explaining variation in amplitude of vibrations induced by airborne noise playbacks (airborne noise experiment) plotted against leaf area, which was the trait that significantly affected the amplitude of vibrations. The amplitude model contained the leaf traits: leaf thickness, leaf area, and water content. Additionally, point on the leaf nested in plant species was included as a random effect with a random intercept. Amplitude is higher on larger leaves. The blue line represents the linear relationship between the fit of the model and leaf thickness

**Figure 8:**
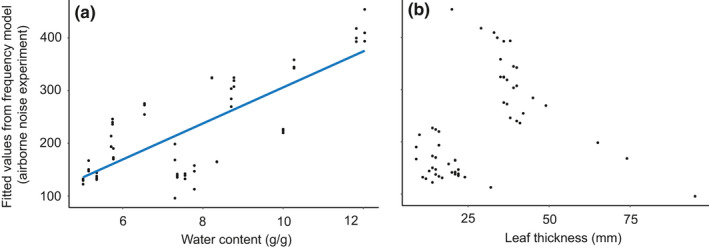
Fitted values from the frequency model explaining variation in peak frequency of vibrations induced by airborne noise playbacks plotted against (a) water content and (b) leaf thickness. The frequency model contained the leaf traits: leaf thickness, leaf area, and water content. Additionally, point on the leaf nested in plant species was included as a random effect with a random intercept. (a) Vibrations recorded on leaves with higher water content had a higher peak frequency. (b) Higher leaf thickness also led to a higher peak frequency. The blue lines represent the linear relationship between the fit of the model and (a) water content and (b) leaf thickness

Leaf area and water content were included in the top models explaining variation in amplitude of vibrations induced by airborne noise playbacks (Appendix S1, Table S5). Leaf area had a significant positive effect on amplitude, with higher amplitude recordings on larger leaves (Figure 7, Table 3). All leaf traits appeared in the top models explaining variation in frequency induced by airborne noise (Appendix S1, Table S5). Leaf thickness and water content had a significant effect on frequency (Figure 8, Table 3), with an increase in both of these traits leading to higher peak frequency recordings.

**Table 3:** Conditional averaged estimates from candidate models testing the effect of leaf traits on the amplitude and peak frequency of vibrations induced by airborne noise playbacks

Parameter	Estimate	*SE*	*z* value	*p* value	95% CI	
					Lower	Upper
Amplitude model						
Leaf area (cm^2^)	0.88388	0.29975	2.949	.00319*	0.2963770	1.4713922
Water content (g/g)	−0.43681	1.51265	0.289	.77276	−3.4015498	2.5279280
Leaf thickness (mm)	−0.03215	0.14415	0.223	.82350	−0.3146789	0.2503756
Frequency model						
Water content (g/g)	30.678	13.218	2.321	.0203*	4.771620	56.583662
Leaf thickness (mm)	−3.402	1.515	2.245	.0248*	−6.371928	−0.431716
Leaf area (cm^2^)	−3.632	4.187	0.867	.3857	−11.839161	4.574371

Note

All models within 4 AICc were considered.

## 4 DISCUSSION

Although vibrational signals and cues probably fall within the least known sensory modality, they are extremely widespread with hundreds of thousands of invertebrate species and many vertebrates relying on them (Cocroft et al., 2014). Vibrational signals and cues are used in a variety of interactions like mating (e.g., white‐lipped frog *Leptodactylus albilabris;* the prairie mole cricket *Gryllotalpa major;* the meadow katydid *Conocephalus nigropleurum* and the treehopper *Ennya chrysura*), predator‐prey (e.g., the Namib Desert golden mole *Eremitalpa granti namibensis;* the sand scorpion *Paruroctonus mesaensis;* the larvae of antlion *Myrmeleon formicarius* and the red‐eyed tree frog *Agalychnis callidryas*), within group (e.g., the leaf cutter ant *Atta sexdens;* treehopper *Umbonia crassicornis* and kangaroo rats of the genus *Dipodomys*) (reviewed in Hill, 2009), and plant‐insect interactions (e.g., *Pieris rapae* and *Arabidopsis thaliana*) (Appel & Cocroft, 2014).

The role of substrate variation in vibrational signal transmission has received increasing attention in recent years (Casas & Djemai, 2002; Casas et al., 2007; Cocroft & Rodriguez, 2005; Cocroft et al., 2006; Čokl et al., 2004, 2005, 2007; Elias et al., 2004; Joyce et al., 2008, 2014; Magal et al., 2000; Polajnar et al., 2012), and there is a general consensus that substrate variation can affect the spectral and temporal characteristics of vibrational signals, potentially affecting animal communication (McNett & Cocroft, 2008). However, little attention has been given to the effect of substrate variation on prey cues. This is particularly interesting in light of the arms race between plants, their herbivores, and the natural enemies of plant herbivores where vibrations have been demonstrated to mediate orientation toward prey (vibrotaxis) by insect parasitoids (Meyhofer & Casas, 1999) and predators (Pfannenstiel et al., 1995). Additionally, a reverse interaction—avoidance behavior in response to semi‐specific vibrational cues produced by foraging predators, has also been shown (Castellanos & Barbosa, 2006). In this study, we investigated the effect of leaf trait variation on the production, transmission, and detectability of chewing cues of a generalist herbivore, the beet armyworm *S. exigua*.

We found the highest amplitude chewing vibrations on sunflower and the lowest on beetroot. These plants differed particularly in their leaf thickness. However, when testing specifically for the effect of leaf traits on chewing vibrations we did not find a clear effect for most traits. Only leaf thickness tended to show a negative relation with amplitude. Caterpillar weight had a highly significant effect on amplitude, with heavier caterpillars producing higher amplitude cues. Moreover, distance from the chewing point to the recording point had a close to significant effect on peak frequency measurements, with lower peak frequency recorded at larger distances. Herbaceous plant tissues act as a low‐pass filter for vibrations (Cocroft et al., 2006; Čokl et al., 2005), so this was an expected result.

Our analysis of the transmission experiment revealed that leaf thickness significantly predicted signal attenuation on the stimulated leaf, with thicker leaves leading to higher attenuation. These finding corroborate the findings on chewing vibrations per plant species, as well as for leaf thickness specifically. Recent similar research also shows a negative correlation between leaf thickness and vibrational noise recorded on the leaf (Li & Kang, 2018). We did not find, however, an effect of leaf traits on attenuation on the adjacent leaves. Unfortunately, we did not measure distance from one leaf to the next adjacent leaf or vein architecture, which might be better predictors of attenuation at that scale. Furthermore, none of the leaf traits we measured significantly predicted variation in peak frequency measurements.

Differences in amplitude within a leaf are particularly important for Branconidae, Eulophidae, and Pteromalidae families of parasitoids, which parasitize hosts that are normally hidden in the substrate (e.g., endophytic Dipteran or Lepidoteran hosts). In these cases, vibrations are usually the main stimuli used (reviewed in Meyhofer & Casas, 1999). For example, Djemai, Casas, and Magal (2004) showed that the parasitoid *Sympiesis sericeicornis* spent more time searching for its leaf‐miner host on a leaf and produced a higher number of oviposition insertions when stimulated with simulated host vibrations. Therefore, for parasitoids or predators searching for their host or prey within leaves (e.g., predators or parasitoids of leaf‐miners), choosing plants with lower leaf thickness may be beneficial as chewing vibrations propagating through the leaf are likely to be higher in amplitude and therefore more likely to be detected.

It is surprising that we did not find clear effects of leaf traits on the amplitude or frequency characteristics of caterpillar chewing vibrations, whereas we did find an effect on the transmission of a synthetic vibrational stimulus. It could be that at the distances at which we recorded chewing vibrations, the effects of leaf traits are not yet detectable, since the effect of the cue‐producing animal had not yet attenuated much and was still dominant over any reflections. It is also possible that leaf traits affect production and transmission differently. Nonetheless, although the effect of leaf thickness on amplitude of chewing cues was not significant, the confidence intervals barely overlapped zero, and leaf thickness showed a similar trend as in the transmission experiment, with higher amplitude cues recorded in plants with the thinnest leaves. Alternatively, the lack of an obvious effect of leaf traits could indicate that there is something happening at the production level. Perhaps caterpillars “prepare” their substrate before chewing it (e.g., via saliva secretions), standardizing the toughness of the plant material they consume, and consequently standardizing the amplitude and frequency characteristics of their chewing cues. Several caterpillar species, including *S. exigua* are known to regurgitate oral secretions on the leaves on which they feed (Alborn et al., 1997; Peiffer & Felton, 2009; Vadassery et al., 2012). Although these oral secretions have been mostly studied for their effect on eliciting plant defenses (Peiffer & Felton, 2009; Vadassery et al., 2012), it is possible that they play an alternative role. A transmission experiment with actual foraging caterpillars where both foraging location and caterpillar weight are controlled for would make an interesting follow up study. Such an experiment would tell us whether the observed lack of effect of leaf traits is because of distance limitation, or whether it is caused at the production level. Another possible factor is leaf geometry, which influences vibrational modes, but the effect is unpredictable and cannot yet be realistically investigated without interfering with the performance of the living plant (de Langre, 2019).

Background noise originating from abiotic sources like wind and rain, or anthropogenic noise from traffic, can interfere with detection of vibratory cues as shown for web‐building spiders (Wu & Elias, 2014). Acoustic noise can be picked up by elastic substrates like plants and continues traveling as vibrational noise within the substrate, which can affect many vibrational interactions, including predator‐prey interactions (Virant‐Doberlet, Kuhelj, Polajnar, & Šturm, 2019). The impact of anthropogenic acoustic noise on communities is thus even more pervasive than commonly recognized. The frequency and intensity of pressure waves, as well as the difference of the acoustic impedances of air and the substrate, affects the penetration of acoustic noise into the substrate (Cremer et al., 2005). We measured the amplitude and frequency of vibrations induced by airborne noise and found, not surprisingly, that leaf area significantly affected the amplitude of vibrations induced by airborne noise, with larger leaves absorbing more noise. This is also consistent with the research of Li and Kang (2018) who found a highly positive correlation between leaf area and leaf vibrations induced by airborne noise. An interesting result was the effect of punch force and water content on the frequency of vibrations induced by airborne noise. Higher water content and higher punch force led to vibrations with higher peak frequency, possibly due to increased stiffness which is associated with higher natural frequencies in finite structures that in turn influence mechanical response to a sound field (Norton & Karczub, 2003).

In conclusion, we found that leaf traits do not affect the amplitude and frequency characteristics of vibrational chewing cues, but they do affect their transmission and possibly their detectability. The discrepancy between the effects of leaf traits on the production of chewing cues and their transmission and detectability is surprising. However, we speculate that “treatment” of plant substrates by caterpillars prior to chewing could explain the lack of differences in amplitude and frequency characteristics across plant species.

In this study, we identified some possible ecologically important linkages between plant and insect strategies mediated by chewing vibrations. Plants may for example evolve thicker leaves to defend themselves against their herbivores, such as caterpillars or other types of invertebrate pests, but by doing so, they may reduce effective use of vibratory cues by the predators and parasites that can eavesdrop on the chewing activity of these herbivores. Focusing on the role of species‐specific leaf traits in plant‐borne vibrations may also provide insight into potential life‐history trade‐offs. Potential leaf trait candidates may include traits related to the architecture, diameter, and internal structure of veins. Leaf surface properties related to epidermal structures and cuticles, rarely measured by plant ecologists, may also provide further traits of interest in this context. Trade‐offs may also be operating across other life‐history domains. For instance, a fast plant‐growth strategy requires investment in leaves that might be more productive, for example, through higher vein density to promote water transport (Brodribb et al., 2007); such a strategy may also lead to greater absorbance of vibration energy, which may also reduce the effectiveness of predators that use vibratory cues of herbivores. In‐depth investigation into the role of plant morphology and physiology in influencing the sensory ecology of primary and secondary consumers will greatly increase our understanding of their complex eco‐evolutionary relationships.

## CONFLICT OF INTEREST

None declared.

## AUTHOR CONTRIBUTIONS

**Estefania Velilla:** Conceptualization (equal); data curation (lead); formal analysis (lead); investigation (lead); methodology (equal); visualization (lead); writing–original draft (equal); writing–review and editing (equal). **Jernej Polajnar:** Conceptualization (equal); investigation (equal); methodology (equal); writing–review and editing (supporting). **Meta Virant‐Doberlet:** Conceptualization (equal); methodology (equal); resources (supporting). **Daniel Commandeur:** Investigation (supporting). **Ralph Simon:** Formal analysis (supporting); software (supporting); visualization (supporting). **Hans J. H. C. Cornelissen:** Conceptualization (equal); methodology (equal); resources (supporting); writing–review and editing (supporting). **Jacintha Ellers:** Conceptualization (equal); supervision (supporting); writing–review and editing (supporting). **Wouter Halfwerk:** Conceptualization (equal); methodology (equal); resources (lead); supervision (lead); writing–original draft (equal); writing–review and editing (equal).

## Supporting information

Appendix S1

## Data Availability

Plant trait measurements, caterpillar chewing vibrations measurements, transmission experiment measurements, airborne noise exposure measurements and all associated R scripts are accessible on Dryad, https://doi.org/10.5061/dryad.ttdz08kw6
