# Supplementary material for: Variation in plant leaf traits affects transmission and detectability of herbivore vibrational cues
Source: Ecol Evol. 2020 Sep 30;10(21):12277–89. doi: 10.1002/ece3.6857 (PMC7663069; doi:10.1002/ece3.6857)
Supplement: Supplementary file 1 — Appendix S1 [file ECE3-10-12277-s001.docx]

**Appendix**

**Table S1.** Candidate models testing the effect of leaf traits on the amplitude and peak frequency measurements from caterpillar chewing vibrations

| **Log(amplitude) from chewing vibrations ~** | **AICc** | **ΔAIC** | **Weight** |
| --- | --- | --- | --- |
| Caterpillar weight + leaf thickness + (1\|species) | 160.15 | 0 | 0.584 |
| Caterpillar weight + (1\|species) | 161.03 | 0.88 | 0.375 |
| **Peak frequency from chewing vibrations ~** |  |  |  |
| Caterpillar weight + distance + leaf thickness + water content + (1\|species) | 773.2 | 0 | 0.568 |
| Caterpillar weight + leaf thickness + water content + (1\|species) | 775.9 | 2.69 | 0.147 |
| Caterpillar weight + distance + leaf thickness + (1\|species) | 776.5 | 2.37 | 0.144 |

**Table S2.** Candidate models testing the effect of leaf traits on the amplitude and peak frequency measurements from the vibrational sweep playbacks on stimulated leaves

| **Attenuation from sweep playbacks ~** | **AICc** | **ΔAIC** | **Weight** |
| --- | --- | --- | --- |
| Leaf thickness + (1\|species/point) | 1093.5 | 0 | 0.544 |
| Leaf thickness + SLA + (1\|species/point) | 1095.6 | 2.07 | 0.193 |
| Leaf thickness + water content + (1\|species/point) | 1095.7 | 2.14 | 0.185 |
| Leaf thickness + SLA + water content + (1\|species/point) | 1097.4 | 3.92 | 0.076 |
| **Peak frequency from vibratory sweep playbacks ~** |  |  |  |
| (1\|species/point) | 1818.7 | 0 | 0.389 |
| SLA + (1\|species/point) | 1820.5 | 1.82 | 0.156 |
| Water content + (1\|species/point) | 1820.7 | 1.98 | 0.144 |
| Leaf thickness + (1\|species/point) | 1820.8 | 2.12 | 0.134 |
| SLA + water content + (1\|species/point) | 1822.6 | 3.94 | 0.054 |
| Leaf thickness + SLA + (1\|species/point) | 1822.6 | 3.95 | 0.053 |

**Table S3.** Candidate models testing the effect of leaf traits on the amplitude and peak frequency measurements from the vibrational sweep playbacks on adjacent leaves

| **Amplitude from vibratory sweep playbacks on adjacent leaf ~** | **AICc** | **ΔAIC** | **Weight** |
| --- | --- | --- | --- |
| (1\|species/point) | 391.7 | 0 | 0.367 |
| Leaf thickness + (1\|species/point) | 392.8 | 1.15 | 0.206 |
| SLA + (1\|species/point) | 394.0 | 2.29 | 0.116 |
| Water content + (1\|species/point) | 394.1 | 2.39 | 0.111 |
| Leaf thickness + SLA + (1\|species/point) | 394.7 | 3.05 | 0.079 |
| Leaf thickness + Water content + (1\|species/point) | 395.3 | 3.6 | 0.060 |
| **Frequency from vibratory sweep playbacks on adjacent leaf ~** |  |  |  |
| (1\|species/point) | 616.3 | 0 | 0.449 |
| Leaf thickness + (1\|species/point) | 618.6 | 2.32 | 0.140 |
| Water content + (1\|species/point) | 618.6 | 2.35 | 0.138 |
| SLA + (1\|species/point) | 618.7 | 2.36 | 0.137 |

**Table S4.** Conditional averaged estimates from candidate models testing the effect of leaf traits on the amplitude and peak frequency measurements from vibrational sweep playbacks on the adjacent leaf. All models within 4 AICc points of each other were considered

|  |  |  |  |  | **95% CI** | |
| --- | --- | --- | --- | --- | --- | --- |
| **Parameter** | **Estimate** | **SE** | ***z* value** | ***p* value** | **Lower** | **Upper** |
| **Amplitude model** |  |  |  |  |  |  |
| Leaf thickness | -9.76930 | 8.42951 | 1.159 | 0.246 | -26.2410072 | 6.7522303 |
| SLA | -0.07192 | 0.12999 | 0.553 | 0.580 | -0.3267079 | 0.1828604 |
| Water content | -0.11257 | 0.75588 | 0.149 | 0.882 | -1.5940643 | 1.3689200 |
| **Frequency model** |  |  |  |  |  |  |
| Leaf thickness | 12.6433 | 44.9853 | 0.281 | 0.779 | -75.5263390 | 100.8128654 |
| Water content | 0.8146 | 3.6230 | 0.225 | 0.822 | -6.2862267 | 7.9154940 |
| SLA | -0.1154 | 0.5806 | 0.199 | 0.842 | -1.253435 | 1.022593 |

**Table S5.** Candidate models testing the effect of leaf traits on the amplitude and peak frequency measurements from vibrations induced by airborne noise playbacks

| **Vibrational noise amplitude induced by airborne noise ~** | **AICc** | **ΔAIC** | **Weight** |
| --- | --- | --- | --- |
| Leaf area + (1\|species/point) | 499.1 | 0 | 0.389 |
| Leaf area + punch force + (1\|species/point) | 500.8 | 1.66 | 0.169 |
| Leaf area + water content + (1\|species/point) | 501.5 | 2.39 | 0.118 |
| Leaf area + leaf thickness + (1\|species/point) | 501.6 | 2.42 | 0.116 |
| **Vibrational noise peak frequency induced by airborne noise ~** |  |  |  |
| Leaf area + leaf thickness + (1\|species/point) | 715.4 | 0 | 0.210 |
| Leaf thickness + water content + (1\|species/point) | 716.4 | 0.94 | 0.131 |
| Leaf area + leaf thickness + water content + (1\|species/point) | 716.5 | 1.12 | 0.120 |
| Leaf thickness + (1\|species/point) | 716.6 | 1.18 | 0.117 |
| Punch force + water content + (1\|species/point) | 716.6 | 1.22 | 0.114 |
| Leaf thickness + punch force + water content + (1\|species/point) | 717.4 | 2.01 | 0.077 |
| Leaf area + leaf thickness + punch force + (1\|species/point) | 717.8 | 2.43 | 0.062 |
| Leaf thickness + punch force + (1\|species/point) | 718.9 | 3.51 | 0.036 |
| Leaf thickness + punch force + water + content + (1\|species/point) | 718.9 | 3.52 | 0.036 |
| Leaf area + punch force + water content + (1\|species/point) | 719 | 3.61 | 0.035 |

**
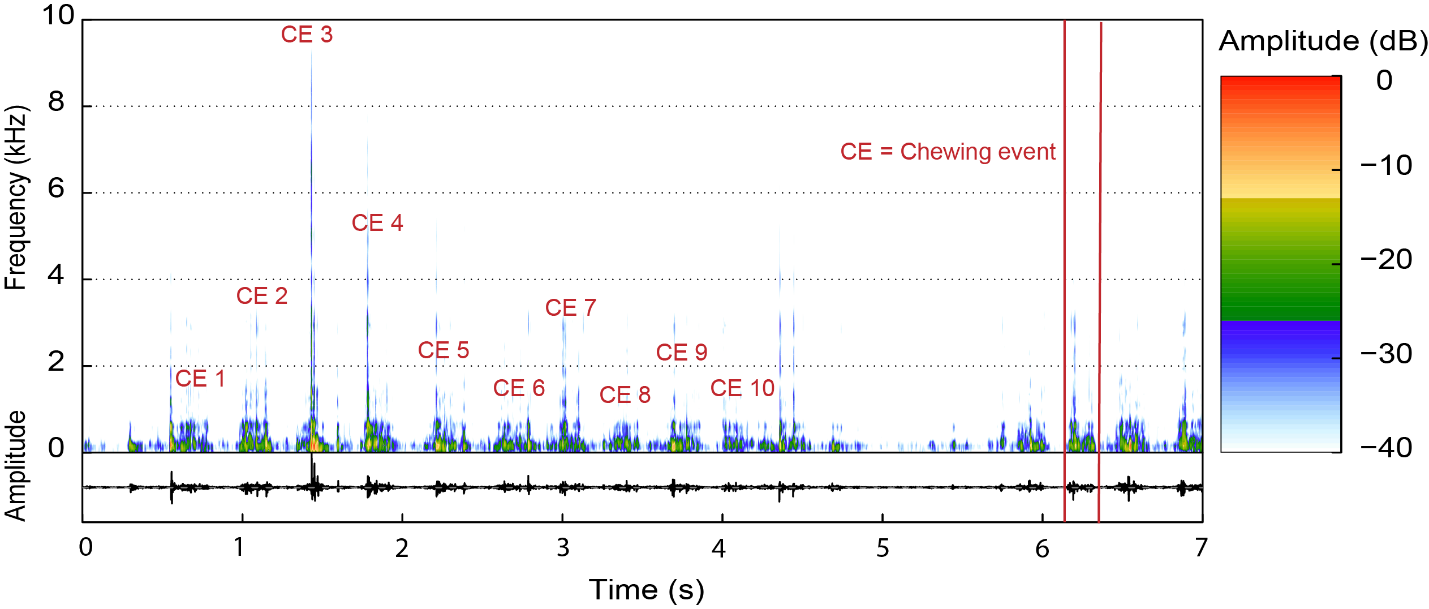
**

**Fig. S1** Example of chewing event selection. Events were selected by hand in Raven Pro 1.5. RMS amplitude was measured from the waveform and peak frequency from the power spectrum, for each selection. For our analysis we took the average RMS amplitude and peak frequency of the 10 selections. This is a recording of Spodoptera exigua foraging on a beetroot plant. CE indicates chewing event
